# Professor David Good, Past President of the World Federation for NeuroRehabilitation: Adapted Interview from the 12^th^ World Congress for NeuroRehabilitation (WCNR), Vienna, 2022

**DOI:** 10.25122/jml-2023-1013

**Published:** 2023-03

**Authors:** Andreea Doria Constantinescu, Alexandra Gherman

**Affiliations:** 1 RoNeuro Institute for Neurological Research and Diagnostic, Cluj-Napoca, Romania

**Interviewer: Andreea Doria Constantinescu**

**Interviewee: Professor David Good** °

° Professor and Former Chair of Neurology at Penn State University, Pennsylvania, United States

Doctor at Milton S. Hershey Medical Center, Hershey, United States

Dr. Good (BS, MD, FASNR) is a Professor Emeritus of Neurology at Penn State College of Medicine. He obtained his MD degree from the University of Wisconsin and received his Neurology training at the University of Minnesota. He has served in academic positions at Southern Illinois University School of Medicine, Wake Forest University, and Penn State College of Medicine, where he was the founding Chair of the Department of Neurology. Professor Good has a long career in neurorehabilitation and has served in several leadership positions, including President of the American Society of Neurorehabilitation and President of the World Federation for Neurorehabilitation (WFNR). He is the author and co-author of multiple abstracts and peer-reviewed papers and has co-edited 3 books on neurorehabilitation. He has lectured widely on neurorehabilitation topics, and his current research focuses on motor recovery following a stroke.

**A.D.C.:** Dear Professor Good, we are here, in Vienna, for the 12^th^ World Congress for NeuroRehabilitation, organised by the World Federation for NeuroRehabilitation. What is your first-hand opinion on this event so far?

**D.G.:** I think the event has been tremendously successful. There has been good attendance, there’s a lot of enthusiasm by the attendees, and the science has been top-rate. The venue in Vienna has also been wonderful. It’s a great time of the year to be in Vienna, so if we have any free time, we can get to enjoy the city a little bit. I think that the set-up at the Convention Centre is very nice; we’ve had very good support, not only from the Convention Centre but from the other staff that we have from EFNR and the WFNR, as well.

**A.D.C.:** Have you participated in any previous editions?

**D.G.:** Yes, all of them, except the first two. This is, actually, the second congress we’ve had in Vienna. We had one, I believe it was in 2010, so this is the second one. I would say that they keep getting better. The only problem was that we had to have a virtual congress during the Covid-19 pandemic. It was supposed to be in Lyon, France, and we, unfortunately, had to have a virtual meeting. But it went fairly well, and we were able to put it all online. I would mention one thing, perhaps this is a good time to mention it - at this meeting, there are, I believe, 1,200 or 1,300 people here on-site, but we also have a few hundred virtual, and that’s really unique. We haven’t done a combination Congress before. And the reason that is important is that there are many people who either can’t come to Vienna or can’t afford to come to Vienna. One of our goals with WFNR is to encourage neurorehabilitation in low- and middle-income countries. Many people from those countries can’t come in person because of the costs. So, having a virtual congress is also very helpful for them.

**A.D.C.:** What is the overarching theme of this year’s congress, from your point of view?

**D.G.:** I think there are several. One is the continuing importance of technology in neuro-rehab; we have an outstanding exhibitors’ area, where there is a great deal of excitement and all sorts of devices and technology, not only computer-based but physical-based technology as well – to assist movement, for example. So, that’s certainly very important. Another important feature are the plenary sessions. For example, the opening plenary session was a *Michael Barnes lecture*, and it was absolutely outstanding. The room was full, the lecture was great. There are ongoing plenary sessions where [the] speakers have been very carefully chosen. So, that’s another highlight, I think, of the meeting. Another important thing is the emphasis on the young members. One of our goals this year was to have a focus on what we call ‘Young WFNR’. They don’t have to be really young, but they should be less than 10 years in either research or practice of neurorehabilitation. It can be an older person but relatively new to the discipline. We have special sessions on that, there’s a workshop, and there are symposia that are dedicated to what we call ‘Young WFNR’ members. And that’s going to be a real focus going forward, too. We also have developed a mentorship program - I had contact with a mentee that was assigned to me today; actually, he contacted me by e-mail. And all of the most senior members of WFNR have been assigned a younger mentee to help guide their careers. So, that has been another focus of congress.

**A.D.C.:** My next question relates to this young new generation. What do you think is the impact of such events on the young generations of specialists in neurorehabilitation?

**D.G.:** These people are the future of neurorehabilitation, and it is important to get them engaged and excited early on in their careers, and I hope that these people will then carry on the traditions and come up with new ideas all the time. When you have grey-haired guys and ladies like me that are doing things all the time – things can get a little bit static – we need new ideas. And the ‘Young WFNR’ could provide those ideas.

**A.D.C.:** You already mentioned the benefits of a hybrid event; with the next question, maybe you can extend it a bit. From your perspective, what is the role of hybrid multidisciplinary events in developing neurorehabilitation research and practice, and what similar avenues are worth exploring?

**D.G.:** That’s a good question. The WFNR is a multidisciplinary organisation. We accept everybody as a member who is interested in rehabilitation, whether they are medical people or physiotherapists or occupational therapists, or speech therapists – everybody is invited to be a member. Over the time that I’ve been in neurorehabilitation, I’ve seen a tremendous amount of multidisciplinary research. Way back, a long time ago, there were only the medics, and the MDs, who were doing the research, and now, everybody is involved in the research; it usually is multidisciplinary – people working together on a project. Just for example, I’ve seen tremendous growth in the number of PhD physiotherapists, who are very active in research, working often with medics and other people as well. Bio-engineers – there’s tremendous research going on with bio-engineering. So, yes, all of these things are very important. And they’ll continue to develop. A lot of it depends on the individual people and the interest they have, and it’s important for people to link up with other people with similar ideas, especially of different disciplines. That makes the research stronger.

**A.D.C.:** What are the main changes in motor recovery after a stroke?

**D.G.:** That’s another very good question. It depends on whether you have a positive attitude about that or a negative attitude. One of the problems that we have with motor recovery is the fact that many people who have a stroke have the destruction of the main motor tracts – from the motor cortex down to the spinal cord. If those tracts are completely destroyed – we call it the cortical-spinal tract, the chances for recovery are limited because those tracts don’t regenerate by themselves. If there is only partial damage, then we can train other areas to be able to help - not to regrow those tracts but to make them more efficient. I think one of the hopes is that we can bypass some of those things. For example, some of the technology that we have now: the brain-computer interface (BCI): people can think about a movement and have a robotic device make the movement. So, essentially, bypass the neural pathways that are destroyed, and you provide an alternate way for the person to move. And I think that’s really exciting for the future. And there are some other things besides the brain-computer interface (BCI). So, I’m hopeful, but I don’t know if we are ever going to be able to regenerate the neural pathways if they are completely destroyed. We have to find other ways around that. There is another point, I guess - if you have a person who does not have complete destruction of the neural pathways and has some residual functions, then it is very important that they have training programs, like physiotherapy programs. And while those are available in developed countries, they aren’t developed all the time in a developing world. For example, if you look at Africa, I’ve seen the figures showing that the number of physiotherapists per 100,000 population is extremely small compared to the developed countries. And so, we have to really work in those parts of the world to get more training for physiotherapists and occupational therapists so they can help the people that have hope for recovery with rehab. And that’s another point I would make, another challenge for motor recovery.

**A.D.C.:** Thank you very much for accepting our invitation and for sharing a bit of your experience in rehabilitation and the history of this congress!

**D.G.:** It’s my pleasure and thank you very much for inviting me!

